# Inhibitory Action of Benzo[*α*]pyrene on Hepatic Lipoprotein Receptors *In Vitro* and on Liver Lipid Homeostasis in Mice

**DOI:** 10.1371/journal.pone.0102991

**Published:** 2014-07-23

**Authors:** Hamed Layeghkhavidaki, Marie-Claire Lanhers, Samina Akbar, Lynn Gregory-Pauron, Thierry Oster, Nathalie Grova, Brice Appenzeller, Jordane Jasniewski, Cyril Feidt, Catherine Corbier, Frances T. Yen

**Affiliations:** 1 Unité de Recherche Animal et Fonctionnalités des Produits Animaux EA3998, Université de Lorraine, Vandœuvre-lès-Nancy, France; 2 Institut National de Recherche Agronomique USC 0340, Vandœuvre-lès-Nancy, France; 3 Laboratory of Analytical Human Biomonitoring, Centre de Recherche Public de la Santé, Luxembourg, Luxembourg; 4 Laboratoire d'Ingenérie des Biomolécules, Université de Lorraine, Vandœuvre-lès-Nancy, France; 5 Institut National de la Santé et de la Recherche Médicale, Vandœuvre-lès-Nancy, France; University of Milan, Italy

## Abstract

**Background:**

Dyslipidemia associated with obesity often manifests as increased plasma LDL and triglyceride-rich lipoprotein levels suggesting changes in hepatic lipoprotein receptor status. Persistent organic pollutants have been recently postulated to contribute to the obesity etiology by increasing adipogenesis, but little information is available on their potential effect on hepatic lipoprotein metabolism.

**Objective:**

The objective of this study was to investigate the effect of the common environmental pollutant, benzo[*α*]pyrene (B[*α*]P) on two lipoprotein receptors, the LDL-receptor and the lipolysis-stimulated lipoprotein receptor (LSR) as well as the ATP-binding cassette transporter A1 (ABCA1) using cell and animal models.

**Results:**

LSR, LDL-receptor as well as ABCA1 protein levels were significantly decreased by 26–48% in Hepa1-6 cells incubated (<2 h) in the presence of B[*α*]P (≤1 µM). Real-time PCR analysis and lactacystin studies revealed that this effect was due primarily to increased proteasome, and not lysosomal-mediated degradation rather than decreased transcription. Furthermore, ligand blots revealed that lipoproteins exposed to 1 or 5 µM B[*α*]P displayed markedly decreased (42–86%) binding to LSR or LDL-receptor. B[*α*]P-treated (0.5 mg/kg/48 h, *i.p.* 15 days) C57BL/6J mice displayed higher weight gain, associated with significant increases in plasma cholesterol, triglycerides, and liver cholesterol content, and decreased hepatic LDL-receptor and ABCA1 levels. Furthermore, correlational analysis revealed that B[*α*]P abolished the positive association observed in control mice between the LSR and LDL-receptor. Interestingly, levels of other proteins involved in liver cholesterol metabolism, ATP-binding cassette transporter G1 and scavenger receptor-BI, were decreased, while those of acyl-CoA:cholesterol acyltransferase 1 and 2 were increased in B[*α*]P-treated mice.

**Conclusions:**

B[*α*]P demonstrates inhibitory action on LSR and LDL-R, as well as ABCA1, which we propose leads to modified lipid status in B[*α*]P-treated mice, thus providing new insight into mechanisms underlying the involvement of pollutants in the disruption of lipid homeostasis, potentially contributing to dyslipidemia associated with obesity.

## Introduction

Obesity has become an increasing problem of public health in the industrialized world [1], [2], and represents a significant risk factor for many pathologies including cardiovascular disease and diabetes. Its origin is complex and multifactorial, where numerous genetic and environmental factors have been shown to contribute to the etiology of obesity. While the adipocyte represents the site of fat accumulation in obesity, the role of the liver as a central station for the reception and delivery of lipids in the form of lipoproteins to the different peripheral tissues should also be considered in investigations of the underlying causes of disrupted lipid status. Indeed, obesity is often associated with dyslipidemia, demonstrated by changes in plasma lipoproteins, including increased levels of triglyceride (TG)-rich lipoproteins (TGRL), and low-density lipoprotein (LDL), and sometimes decreased levels of high density lipoprotein (HDL) [3]. Increased TGRL in obesity has been attributed to overproduction of VLDL [4], but elevated postprandial lipemia in obese subjects suggests decreased ability to remove intestinally-derived chylomicrons. The removal of lipoproteins from the circulation is mediated by hepatic lipoprotein receptors, including the LDL-receptor (LDL-R), well-known for its key role in the regulation of cholesterol metabolism [5], but which also participates in the removal of TGRL. The lipolysis stimulated lipoprotein receptor (LSR) has recently been shown by our laboratory and others to play an important role in the removal of TGRL during the postprandial phase [6]–[8]. Interestingly, if these receptors are deficient (LSR^+/−^) or lacking (LDL-R^-/-^) in mice, the animals exhibit not only changes in plasma lipid levels, but also significantly higher weight gain when placed under high-fat diets as compared to wild-type littermates [9], [10]. LSR^+/-^ mice shown to have 50% reduced LSR expression and to display elevated postprandial lipemia and reduced lipid clearance also develop obesity with age, even if maintained on a standard diet [10], therefore suggesting that changes in lipoprotein receptor status leading to dyslipidemia may also increase disposition towards excess weight gain, and thus increased propensity towards developing obesity.

Recent attention has focused on persistent organic pollutants (POP) as potential environmental factors involved in the etiology of obesity, due in part to epidemiological studies demonstrating a correlation between exposure to pollutants and increased risk or incidence of obesity [11], [12] as well as studies demonstrating their role as endocrine disruptors leading to increased adipogenesis [13], [14]. Among them includes the polycyclic aromatic hydrocarbon (PAH) benzo[*α*]pyrene (B[*α*]P), which is a common pollutant that is produced during incomplete combustion of organic material including wood, coal, diesel, tobacco [15]-[17], and is also present in foods due to cooking processes including frying, smoking and grilling [18]. Levels of B[*α*]P in the circulation have been shown to be correlated to body mass index (BMI) in a population study [19]. We have shown that B[*α*]P can exert inhibitory effects on epinephrine-induced lipolysis in the adipocyte, and reported that mice exposed to this pollutant exhibit higher weight gain as compared to control mice [20]. Also, the carcinogenic properties of this environmental pollutant has already been demonstrated where reactive dihydrodiol epoxide derivatives of B[*α*]P metabolites derived from the cytochrome P450 system bind covalently to DNA, leading to adduct formation and subsequent tumors [21]–[23]. Further, B[*α*]P serves as ligand with the aryl hydrocarbon receptor (AHR) [24], [25], shown to be rate-limiting step in B[*α*]P-induced tumor formation [26].

While the carcinogenic aspect of this pollutant has been well-documented, little is known on its potential effect on lipid and lipoprotein metabolism. This pollutant circulates in the blood associated with lipoproteins [27], [28], can penetrate cells *via* biological membranes [29], [30] and subsequently accumulate not only in the lipophilic adipose tissue and mammary fat, but also in other tissues including the liver and kidney [31]. This led us to question if B[*α*]P could disrupt lipid homeostasis *via* an effect on lipoprotein metabolism in the liver. Also, although B[*α*]P may be involved in regulating expression of multidrug resistance transporters [32], no information is available on its potential effect on ATP-binding cassette (ABC) proteins including ABCA1 which is involved in cholesterol efflux from the liver. In this study, we investigate and show evidence for the effect of B[*α*]P on hepatic lipoprotein receptors LDL-R and LSR, and on ABCA1, using both cell and animal models.

## Materials and Methods

### Materials, antibodies and standards

Chemicals and reagents were purchased from Sigma-Aldrich (St Quentin Fallavier, France) unless otherwise indicated. Cell culture media and supplements were obtained from Invitrogen (Alfortville, France). Rabbit anti-LDL-R and anti-LSR antibodies were obtained from Abcam (Paris, France) and Sigma-Aldrich, respectively. Mouse anti-ABCA1 antibodies were purchased from Millipore (Darmstadt, Germany), and goat anti-ACAT1 and anti-ACAT2 antibodies from Santa Cruz Biotechnology (Heidelberg, Germany), respectively. Antibodies recognizing SR-BI or ABCG1 were obtained from Novus Biologicals (Cambridge, United Kingdom). Secondary anti-rabbit and anti-mouse peroxide-conjugated IgG were acquired from Cell Signaling Technology (Boston, MA). Anti-apolipoprotein (apo)B and anti-apoE antibodies, as well as rabbit peroxidase-conjugated anti-goat IgG were obtained from Santa Cruz Biotechnology. B[*α*]P-*d_12_* **,** 1-OH-benz[*α*]anthracene-*^13^C_6_* and all B[*α*]P metabolites (including 1-, 2-, 3-, 4-, 5-, 6-, 7-, 8-, 9-, 10-, 11-, 12-OH-B[*α*]P, 4,5-diol- B[*α*]P cis, 7,8-diol-B[*α*]P cis, 7,8-diol-B[*α*]P trans, 9,10-diol-B[*α*]P trans) investigated in this study were purchased in powder form from MRI Global (Kansas City, MO, USA). B[*α*]P was purchased in powder form from Sigma-Aldrich. B[*α*]P standard, internal standards and OH-B[*α*]P standard stock solutions were prepared in acetonitrile (10 mg/L). Working solutions were prepared in acetonitrile by successive ten-fold dilutions at concentration ranging from 1000 µg/L to 10 µg/L and were stored at −20°C.

### Cell culture studies

The mouse Hepa1–6 liver cell line (DSMZ, Brunswick, Germany) was maintained in Dulbecco's modified Eagle Medium containing 10% fetal bovine serum and 1 mM glutamine [10]. Cells were seeded in 12-well or 24-well plates and used after 48 h at 80–90% confluence. On the day of the experiment, cells were washed in phosphate-buffered saline (PBS), and then incubated at 37°C in 95% air, 5% CO_2_ environment for the indicated times with 0.1 or 1 µM B[*α*]P in 1% (v/v) DMSO; control cells were treated with vehicle (1% (v/v) DMSO). The proteasome inhibitor lactacystin was prepared in DMSO and incubated with cells, with 1% (v/v) DMSO used as control. In experiments using chloroquine, this lysosomal inhibitor (25 µM) was first pre-incubated with cells 2 h at 37°C, before a second incubation of 1 h with 0.1 µM B[*α*]P or 1% (v/v) DMSO. At the end of the incubation periods, cells were then placed on ice and washed 2 times with ice-cold PBS. Cell lysates were recovered using ice-cold radioimmunoprecipitation assay buffer containing anti-proteases [10], followed by centrifugation at 13,000 x g for 30 min at 4°C. Protein was measured in the recovered supernatants using BCA assay (Thermo Scientific, Courtaboeuf, France).

#### Cell viability and metabolic activity assays

In preliminary studies, viability of Hepa1-6 cells was determined using the MTT [3-(4,5-dimethylthiazol-2-yl)2,5-diphenyl tetrazolium bromide] assay [33]. Briefly, Hepa1-6 cells were incubated with filtered (0.22 µM) MTT (67 µg/mL, final concentration) for 20 min at 37°C, followed by incubation at room temperature with DMSO (50% v/v, final concentration) with gentle agitation for 10 min to dissolve the formazan crystals; absorbance was measured at 570 nm. A second method was used to assess cell viability and metabolic activity [34]. Hepa1-6 cells were incubated for 30 min at room temperature with 2 µM calcein-AM. After washing with PBS, cells were incubated for 15 min at room temperature with 1% (v/v) Triton X-100 with gentle agitation. Fluorescence emission intensity was measured (λemission = 530 nm and λexcitation = 485 nm).

Cell viability was also measured using the trypan blue exclusion assay. Aliquots of cells in serum-free medium were diluted with equal volumes of 0.4% (w/v) trypan blue. After 3 min incubation at room temperature, unstained and stained cells were counted in a hemacytometer.

#### Immunoblot analysis

Identical amounts of membrane protein or cell lysate (15-20 µg) were separated on 10% SDS-PAGE gels, followed by transfer to nitrocellulose membranes. Loading was systematically verified using Red Ponceau staining. Immunoblotting was performed as previously described using the different antibodies as indicated [8], [10]. For cell lysates, β-tubulin was used as loading control. Protein bands were revealed by chemiluminescence (GE Healthcare, Orsay, France) using a peroxidase-conjugated secondary antibody and a chemiluminescence kit (GE Healthcare), followed by imaging on a Bio-Rad Fusion FX5 (Vilber Lourmat, France). Densitometric analysis was performed using ImageJ software.

### *In vivo* study

Adult 10 week-old male C57Bl/6J mice weighing 20–22 g were obtained from Charles River Laboratories (L'Arbresle, France) and housed in temperature-regulated (20°C), ventilated cabinets with a 12 h light, 12 h dark cycle (8AM to 8PM) in a certified animal facility. Animals were acclimated in this controlled environment for 1 week prior to the study with a normal rodent chow diet and water *ad libitum* in a room with a mean temperature of 21–22°C and relative humidity of 50 ± 20%.

The study protocols for animal handling and experiments were authorized by the Department for the Protection of Populations (DDPP, Meurthe et Moselle, authorization N° 54-547-24) and in accordance with the European Communities Council Directive of 2010/63/EU. All efforts were made to minimize suffering.

B[*α*]P was solubilized in physiological saline solution containing 5% DMSO and 1% methyl carboxy cellulose, and injected *i.p.* into mice at 8AM every 48 h at a dose of 0.5 mg/kg [20]. The first and last injections were on day 1 and day 15, respectively. The 11-week old animals were selected randomly for each group. Control animals received vehicle (n = 9 per group). Body weight and food intake of individually housed animals were measured at day 0, before every injection, and on day 16. On day 0 and day 16, animals were fasted for 4 h, and lightly anesthetized with isoflurane before blood sampling by submandibular bleeding. Blood (100 µL) was directly placed into tubes containing EDTA on ice, and plasma was obtained by centrifugation at 13,000 x *g* at 4°C for 10 min. Samples were stored for analysis at −20°C. On day 16, animals were exsanguinated by cardiac puncture. Liver, epididymal fat pads of adipose tissue, and gastrocnemius muscle were rapidly dissected, rinsed in physiological saline, and snap frozen in liquid N_2_ for storage at −80°C. Liver total membranes were prepared as described previously [35].

#### Analysis of lipoprotein profiles

Plasma samples of 4 mice from each group were pooled (210 µL total) and then added to 290 µL of 30 mM phosphate buffer containing 150 mM NaCl, 1 mM EDTA, and 0.02% sodium azide, pH 7.4. This was applied (0.2 mL/min) to a Superose 6 10–300 GL column (GE Healthcare) equilibrated with the same buffer. Fractions of 500 µL were collected and then analyzed for total cholesterol (TC) and triglyceride (TG) content using the enzymatic kits as described previously [8].

#### Biochemical determinations

Lipids [(TG, TC, phospholipids (PL)] of plasma, tissue lipid extracts, and fractions from lipoprotein profiles were analyzed as previously described [8] using colorimetric enzymatic kits (Biomerieux, Craponne, France) according to the manufacturer's instructions. A serum control (Unitrol) was included with each assay performed.

#### Real-time PCR analysis

Cell pellets (1–2×10^6^ cells) or frozen liver samples (40-60 mg) were homogenized in QIAzol Lysis reagent (Qiagen, Courtaboeuf, France), according to manufacturer's instructions. Total RNA was extracted using RNeasy lipid tissue minikit (Qiagen); the integrity of the RNA was verified by the presence of 28S and 18S bands on agarose gels. Ten micrograms of total RNA was used for RT from which 500 ng was used for real-time PCR, as described previously [8]. For LSR and LDL-R, reactions were prepared using the Applied Biosystems (Foster City, CA, USA) SYBR Green PCR Master Mix and then performed on the StepOnePlus real-time PCR system (Applied Biosystems). Real-time PCR analysis for mouse ABCA1 was performed using a validated Taqman assay (Mm00442646_m1) obtained from Applied Biosystems. Relative expression calculations and statistical analyses were performed using the Relative Expression Software Tool (REST) 2009.

### Ligand blot studies

Rat liver plasma membranes were prepared as previously described [36]. LDL (1.025< density (*d*) <1.055 g/mL) and VLDL (*d*<1.006 g/mL) were isolated from pooled human plasma [8]. Ligand blots on solubilized protein from rat liver plasma membranes were performed as previously described for LSR [35] and LDL-R [37]. Solubilized membrane protein from rat hepatocyte were separated on a 10% SDS-PAGE gel, followed by transfer to nitrocellulose. After blocking with 3% BSA, nitrocellulose strips were incubated for 30 min at 37°C with 0.8 mM oleate in the presence of 0.1 M phosphate buffer, 350 mM NaCl and 2 mM EDTA (pH 8.0) as described previously for optimal binding of lipoprotein to LSR [35]. For the LDL-R, the binding buffer used was 50 mM Tris-HCl, pH 8 containing 2 mM CaCl_2_, 50 mg/mL of BSA and 90 mM NaCl [37] since binding of LDL-R to its ligand is Ca^2+^-dependent, unlike LSR. VLDL and LDL were preincubated at room temperature for 30 min in the absence or presence of 0, 1, or 5 µM B[*α*]P. The nitrocellulose strips were then incubated for 1 h at 37°C with 20 µg/mL B[*α*]P-VLDL or B[*α*]P-LDL protein, maintaining the same concentrations of B[*α*]P. Following washes with PBS containing 0.5% Triton X-100 for LSR, or 50 mM Tris-HCl, pH 8 containing 2 mM CaCl_2_, 5 mg/mL of BSA and 90 mM NaCl for LDL-R, strips were incubated with rabbit anti-apoB and apoE IgG, followed by secondary conjugated antibodies to detect bound lipoprotein.

### GC-MS/MS Analysis of B[*α*]P and its metabolites in blood

Samples (100 µL) were supplemented with 20 µL of 1-OHbenz[*α*]anthracene-^13^C_6,_ (at 1 mg/mL) and 10 µL of B[*α*]P-*d_12_* (at 1 mg/mL) as internal standards and adjusted to pH 5.7 with 200 µL of 1 M sodium acetate buffer. The hydrolysis, extraction and purification procedures were carried out in accordance with the analytical method recently described by Peiffer *et al*. [38]. Plasma extracts were reconstituted in 25 µL of MSTFA (N-methyl-N-(trimethylsilyl) trifluoroacetamide, Derivatization of target analytes was completed after 30 min at 60°C, and 2 µL of the extract were injected into the GC–MS/MS system. Analyses were carried out with an Agilent 7890A gas chromatograph equipped with a HP-5MS capillary column (30 m, 0.25 mm i.d., 0.25 µm film thickness), coupled with an Agilent 7000B triple quadrupole mass spectrometer operating in electron impact ionization mode and an Agilent CTC PAL autosampler. Analytical conditions used for chromatography and MS/MS detection were as previously described [39]. Calibration curves were performed using plasma specimens supplemented with increased concentration levels of B[*α*]P and of their metabolites from 0 to 125 ng/mL of plasma. Limits of quantification (LOQs) were evaluated at 0.25 ng/mL of plasma for B[*α*]P, ranged from 0.1 to 1.0 ng/mL for monohydroxylated- and dihydroxylated- forms of B[*α*]P.

### Dynamic light scattering (DLS) and Zeta potential measurements

DLS and Zeta potential measurements were performed with a Zetasizer Nano ZS (Malvern, England) equipped with a 532 nm frequency doubled DPSS laser, a measurement cell, a photomultiplier and a correlator. Scattering intensity was measured at a scattering angle of 173° relative to the source using an avalanche of photodiode detector. The software used to collect and analyse the data was the Zetasizer Software version 6.34 from Malvern.

One mL of LDL or VLDL (refractive index 1.47; concentration of 20 µg protein/mL in Tris 0.2 mM) was measured in a clear disposable Zeta cell (DTS1060 Malvern). The measurements were made at a position of 5.50 mm and 2.00 mm from the cuvette wall for the DLS or Zeta potential measurements, respectively, with an automatic attenuator selection and at a controlled temperature of 25°C. This setup allows considerable reduction of the signal due to multiple scattering events and enables working in slightly turbid media. Intensity autocorrelation functions were analysed by CONTIN algorithm in order to determine the distribution of translational *z*-averaged diffusion coefficient of particles, D_T_ (m^2^. s^-1^). The D_T_ parameter is related to the hydrodynamic radius (R_h_) of particles through the Stokes-Einstein relationship: *D_T_  =  k_B_T/6πηR_h_*, where *η* is the solvent viscosity (Pa.s), *k*_B_ is the Boltzmann constant (1.38×10^−23^ N.m.K^−1^), *T* is the absolute temperature (°K) and *R_h_* (m) is the equivalent hydrodynamic radius of a sphere having the same diffusion coefficient than the particles. For each sample, 15 runs of 70 s were performed with three repetitions. Approximate Zeta Potential measurements were obtained from the Smoluchowski equation and the electophoretic mobility values determined using an automatic voltage selection. For each sample, 20 runs were performed with three repetitions.

### Statistical analyses

All results are shown as mean ± SEM, unless otherwise indicated. Statistical differences were tested using one-way or two-way ANOVA, or Student's *t* test as indicated; statistical significance was considered as *P*<0.05. Correlations were evaluated using Pearson or Spearman rank correlation coefficients.

## Results

### Effect of B[*α*]P on protein levels and mRNA expression of LDL-R, LSR and ABCA1 in mouse Hepa1-6 cells

**Figure 1:**
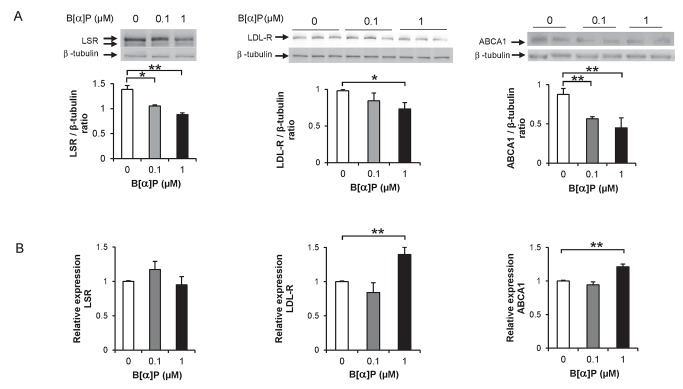
Effect of B[*α*]P on LSR, LDL-R and ABCA1 protein and mRNA levels in Hepa1-6 cells. **A)** Hepa1-6 cells were incubated at 37°C for up to 2 h in the absence or presence of the indicated concentrations of B[*α*]P, after which cell lysates and were prepared as described in Methods. Representative immunoblots (for LSR and ABCA1, results of 1-h incubation are shown; for LDL-R, results for 2-h incubation are shown) and corresponding densitometric analyses using β-tubulin as internal control are shown (one-way ANOVA, *P<0.05, **P<0.01, as compared to 0 µM B[*α*]P, n = 3 different cell preparations using duplicate or triplicate wells). **B)** mRNA levels of LSR, LDLR and ABCA1 were determined in cells incubated with the indicated concentrations of B[*α*]P using real-time PCR, as described in Materials and Methods. Results are shown (n = 4 in triplicate) of LSR, LDLR and ABCA1 mRNA expression relative to HPRT, used as reference housekeeping gene. It should be noted that there was no significant changes in HPRT expression under the different conditions (**P<0.01 as compared to 0 µM B[*α*]P).

We first sought to determine the effect of B[*α*]P on LSR, LDL-R in mouse Hepa1-6 cells, which have been shown previously to express both of these lipoprotein receptors [8]. In preliminary studies in which cells were incubated 1 h at 37°C with increasing concentrations of B[*α*]P from 0.1-100 µM, cell viability, mitochondrial and metabolic activities were not significantly affected, using 3-(4,5-dimethylthiazol-2-yl)-2,5-diphenyltetrazolium bromide (MTT), calcein, and trypan blue as read-outs (Figure S1). Since we had previously observed that this pollutant displayed inhibitory action on epinephrine-induced lipolysis in adipocytes at concentrations <1 µM [20], experiments were performed using similar concentrations. Immunoblots of the lysates from Hepa 1-6 cells incubated at 37°C with 0.1 and 1 µM of B[*α*]P revealed that B[*α*]P induced a significant decrease in the levels of LSR and LDL-R in a dose-dependent manner (Fig. 1A, left and middle panels). For LSR (Fig. 1A, left panel), two LSR bands are typically observed, corresponding to the α (or α') and the β LSR subunits [40]; both subunits were decreased after 1 h incubation with B[*α*]P (Fig. 1A, left panel), while for LDL-R a 2-h incubation was necessary to observe a significant B[*α*]P-induced decrease of this receptor (Fig. 1A, middle panel). Similarly to LSR, ABCA1 protein levels were significantly decreased after 1 h incubation of cells with B[*α*]P (Fig. 1A, right panel). No significant change was observed in the β-tubulin loading control, indicating that these effects did not appear to be related to the general state of the cells.

A second set of cells were treated similarly, from which mRNA was isolated to perform qPCR analysis. Results presented in Figure 1B revealed no significant change in LSR expression relative to the housekeeping gene HPRT, nor for that of LDL-R and ABCA1 in cells incubated in the presence of 0.1 µM B[*α*]P. A small but significant increase in relative expression of LDL-R and ABCA1 was observed when B[*α*]P concentrations were increased to 1 µM (Fig. 1B, middle and right panels) contrary to the decreased protein levels (Fig. 1A). Nonetheless, the results showed that the decrease in protein levels of LSR, LDL-R and ABCA1 was not due to decreased transcription.

### Inhibition of B[*α*]P effect by lactacystin in cells

**Figure 2:**
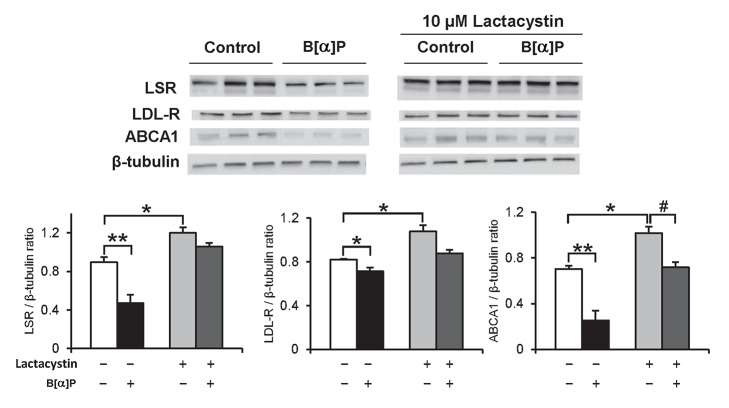
Lactacystin effect on B[*α*]P-induced decrease of LSR, LDL-R and ABCA1 protein levels in Hepa1-6 cells. Hepa1-6 cells were preincubated for 1 h at 37°C with 10 µM lactacystin, followed by 1-h incubation with 0.1 µM B[*α*]P with lactacystin still maintained in the cell medium. Immunoblots were performed on cell lysates, to detect LSR, LDL-R and ABCA1, and are shown with corresponding densitometric analyses (two-way ANOVA, *P<0.05, **P<0.01, *vs* cells incubated in absence of lactacystin and B[*α*]P; # *P*<0.05 *vs* cells incubated with lactacystin alone, n = 3 different wells per treatment).

The lack of significant decreases of mRNA levels in Hepa1-6 cells led us to question if the B[*α*]P-induced effect could have occurred by increasing catabolism, rather than by inhibition of the synthesis of the proteins affected. Ubiquitin-mediated proteolysis in the proteasome is intimately involved in the removal of numerous cellular proteins, and little is known regarding PAH's potential involvement in the proteasome pathway. Cells were exposed to the proteasome inhibitor lactacystin for up to 24 h, and immunoblots revealed that LSR, LDL-R and ABCA1 protein levels in Hepa1-6 cells were all significantly increased after 2 h incubation in the presence of lactacystin (Figure S2). Another set of cells were then treated 1 h with 10 µM lactacystin, followed by addition of 0.1 µM B[*α*]P and an additional incubation of 1 h. Results of the immunoblots confirmed the B[*α*]P-induced decrease of LSR, LDL-R and ABCA1, as well as their increased protein levels in the presence of lactacystin (Fig. 2). In cells pre-incubated with lactacystin, protein levels of LSR and LDL-R were slightly lower when B[*α*]P was present, but did not reach statistical significance as compared to cells pre-incubated with lactacystin in absence of B[*α*]P. The B[*α*]P-induced decrease of ABCA1 in the presence of lactacystin was significant as compared to cells incubated with lactacystin alone. However, this decrease was 2-fold lower as compared to the effect of B[*α*]P on cells in the absence of lactacystin. These data suggest therefore that lactacystin prevented the B[*α*]P-induced decrease of LSR, LDL-R and ABCA1 protein levels, thus suggesting that the effect of this pollutant was mediated through increased degradation in the proteasome.

**Figure 3:**
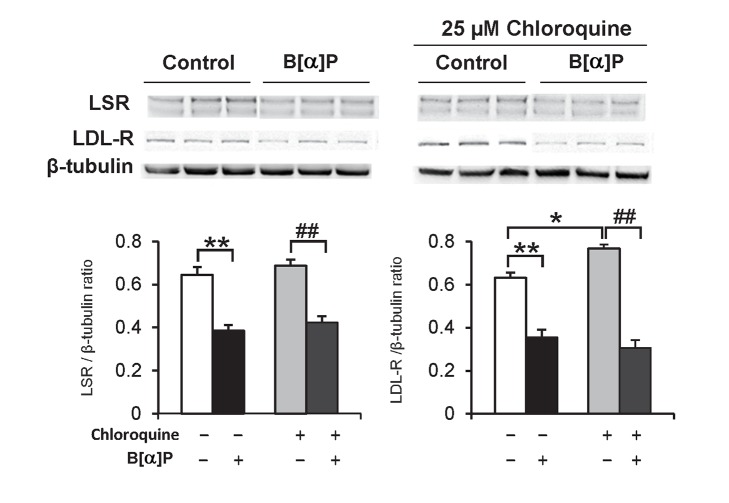
Chloroquine effect on B[*α*]P-induced decrease of LSR, LDL-R protein levels in Hepa1-6 cells. Hepa1-6 cells were preincubated for 2 h at 37°C with 25 µM chloroquine, followed by 1-h incubation with 0.1 µM B[*α*]P with chloroquine still maintained in the cell medium. Immunoblots were performed on cell lysates, to detect LSR and LDL-R, and are shown with corresponding densitometric analyses (two-way ANOVA, *P<0.05, **P<0.01, *vs* cells incubated in absence of chloroquine and B[*α*]P; ## *P*<0.01 *vs* cells incubated with chloroquine alone, n = 3 different wells per treatment).

Once internalized by endocytosis, ligand-bound receptors can be eventually degraded in the lysosome. We performed experiments similar to those described for lactacystin, using chloroquine instead to inhibit lysosomal activity. Results of immunoblots revealed a small but significant increase in LDL-R, but not LSR in cells incubated with chloroquine alone (Fig. 3). B[*α*]P-induced decrease of both LSR and LDL-R remained identical, either in the absence or presence of chloroquine (Fig. 3), suggesting that the observed B[*α*]P-mediated effect was not due to increased degradation in the lysosome.

In view of these changes, we next sought to determine the effect of B[*α*]P *in vivo*. We had previously established that mice treated with B[*α*]P displayed significantly higher weight gain as compared to controls [20]. The same experimental conditions were therefore used to determine the effect of B[*α*]P on hepatic lipid and lipoprotein metabolism.

### Effect of B[*α*]P on plasma and tissue lipids

**Figure 4:**
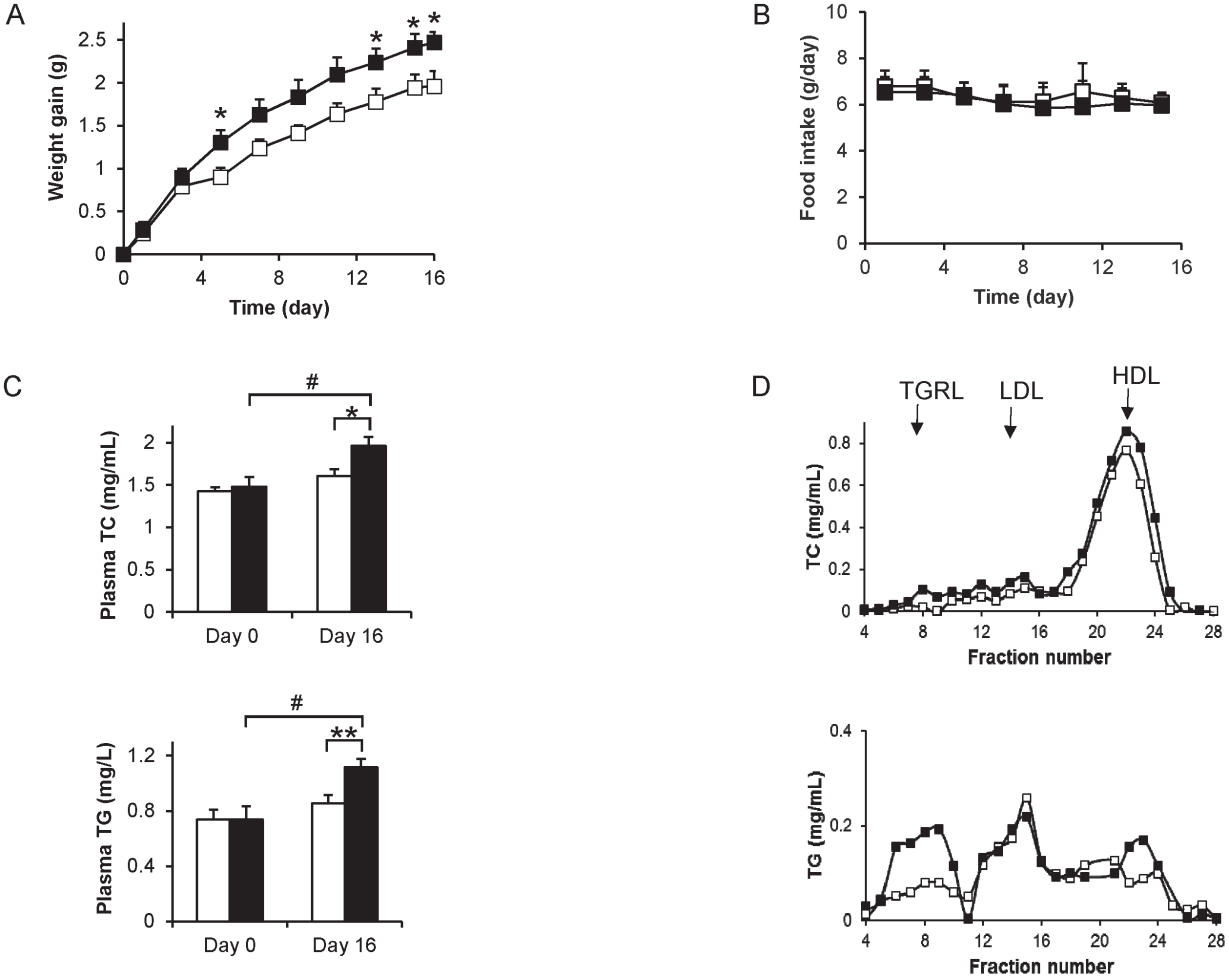
B[*α*]P effect on (A) body weight gain, (B) food intake, (C) plasma lipids, (D) lipoprotein profile in mice. Male 11 week-old C57BL/6J mice were *i.p.* injected every 48 h with vehicle alone (□, n = 9) or vehicle containing 0.5 mg/kg B[*α*]P (▪, n = 9) from day 1 to day 15 and monitored for (**A**) weight gain (two-way ANOVA, *P<0.05 B[*α*]P group as compared to control group), and (**B**) food intake. **C**) Plasma total cholesterol (TC) and triglycerides (TG) were measured on Day 0 and Day 16 in plasma of 4-h fasted animals (two-way ANOVA*P<0.05, **P <0.01 B[*α*]P Day 16 *vs* control Day 16, # *P*<0.05; B[*α*]P Day 16 *vs* B[*α*]P Day 0). **D**) Lipoprotein profiles were obtained from pooled plasma samples obtained on day 16 using gel filtration chromatography as described in Materials and Methods. Fractions were analyzed for TC (upper panel) and TG (lower panel) content. The elution of TG-rich lipoproteins (TGRL), LDL and HDL are indicated by arrows.

Eleven-week old C57BL/6J male mice on a standard diet were injected *i.p.* with 0.5 mg B[*α*]P/kg/48 h (mice were used one week after delivery following a 1 week quarantine). As reported previously [20], body weight increased in both groups continuously during the treatment period (Fig. 4A). At the end of the experimental period, B[*α*]P-treated mice exhibited 26% greater weight gain as compared to controls (*P*<0.03). Monitoring of food intake revealed no detectable difference between the control and experimental groups (Fig. 4B). Plasma levels of B[*α*]P were measured on day 16, and found to be significantly higher in B[*α*]P-treated animals as compared to controls (0.38±0.04 ng/mL and 0.69±0.09 ng/mL for control and B[*α*]P-treated animals, respectively, *P*<0.05). With regards to B[*α*]P metabolites, none were detected in plasma of both control and B[*α*]P-treated mice.

Analysis of plasma after a 4-h fasting period revealed that both plasma TC and TG significantly increased (32 and 51%, respectively) between day 0 and day 16 in animals treated with B[*α*]P, whereas there was no significant difference in mice treated with the vehicle (Fig. 4C). Consequently, the levels of plasma TC and TG were higher (22 and 31%, respectively), in the B[*α*]P-treated mice as compared to controls on day 16. The analysis of lipoprotein profiles from pooled plasma samples using gel filtration chromatography revealed a slight increase of cholesterol levels in the fraction containing TGRL (VLDL and chylomicrons) (Fig. 4D, upper panel), as well as that containing HDL. Since TG is the major lipid component of VLDL lipoproteins, we also measured TG in the fractions and observed an increase primarily in the fractions containing TGRL as compared to those containing LDL or HDL (Fig. 4D, lower panel). This suggests that the observed increase in plasma TG was most likely due to an increase in the TGRL fraction in plasma of B[*α*]P-treated animals.

Mice were sacrificed on day 16 and liver, adipose tissue and skeletal muscle were removed for lipid composition analysis. A small, but significant increase in liver TC content was observed in the B[*α*]P group compared with controls (Table 1). Since liver PL also increased, although not significantly, this may explain the similar TC/PL ratios in both groups. A significant increase (29%) in TG content of adipose tissue of B[*α*]P-treated mice was observed, as compared to those of control animals (Table 1), consistent with the increased fat mass observed in B[*α*]P-treated animals [20]. TC content of adipose tissue, although a minor component in adipose tissue compared to TG, was also significantly increased in mice treated with B[*α*]P. This was similarly the case if expressed as ratios relative to PL in adipose tissue. If it is assumed that PL as a major cell membrane constituent represents an estimate of cell number, this would suggest increased lipid content relative to the number of adipocytes. No detectable difference in lipid content was observed in skeletal muscle.

**Table 1:** Tissue lipid content of control and B[*α*]P-treated mice.

	Liver		Adipose Tissue		Skeletal muscle	
	Control^a^	B[*α*]P^a^	Control^a^	B[*α*]P^a^	Control^a^	B[*α*]P^a^
	(µg/mg dry weight)					
**TC**	4.4±0.14	5.1±0.21*	0.3±0.02	0.5±0.07*	1.8±0.16	1.9±0.07
**TG**	9.3±0.51	9.6±0.69	3.5±0.22	4.5±0.49*	4.1±0.26	4.4±0.23
**PL**	18.2±0.61	19.9±0.96	2.6±0.30	2.2±0.30*	16.9±0.52	15.6±0.85
	(ratio)					
**TC/PL**	0.24±0.01	0.26±0.01	0.14±0.02	0.24±0.02**	0.10±0.01	0.11±0.004
**TG/PL**	0.52±0.04	0.50±0.06	1.50±0.20	2.23±0.25**	0.24±0.01	0.26±0.01

a mean ± SEM (n = 9 for each group).

*P<0.05, **P<0.01, as compared to control group.

**Figure 5:**
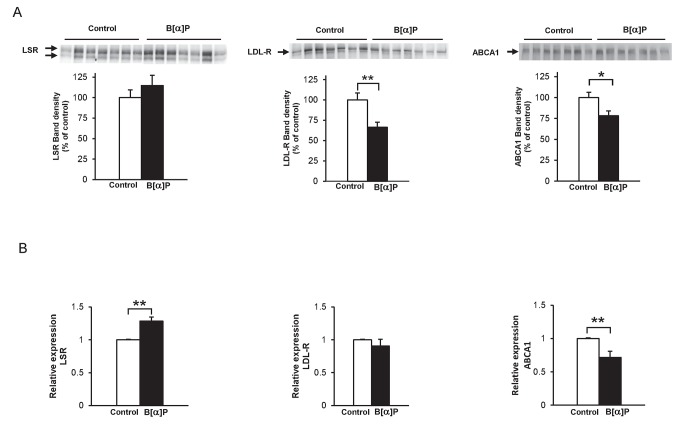
Effect of B[*α*]P on mouse hepatic LDL-R, LSR, and ABCA1 protein and mRNA levels. **A)** Immunoblots to detect LDL-R, LSR, and ABCA1 were performed on protein solubilized from liver total membranes isolated from control and B[*α*]P-treated mice. Blots are shown with corresponding densitometric analysis (Student's t-test, *P<0.05, **P<0.01 B[*α*]P as compared to control groups, n = 7 for each group). **B)** mRNA levels of liver LSR, LDLR and ABCA1 isolated from control and B[*α*]P-treated mice were determined using real-time PCR, as described in Materials and Methods. Results are shown for LSR, LDLR and ABCA1 mRNA expression relative to HPRT, used as reference housekeeping gene. There was no significant changes in HPRT expression under the different conditions (**P<0.01 as compared to control mice; triplicate determinations of n = 4 per group).

Immunoblots on protein solubilized from liver total membranes revealed that protein levels of both LDL-R and ABCA1 were significantly decreased (34% and 22%, respectively) in B[*α*]P-treated mouse liver membranes as compared to those of controls (Fig. 5A, middle and right panels), qPCR analysis revealed that mRNA levels were modified in a similar manner (Fig. 5B, middle and right panels), although the difference was only significant for ABCA1. The increase in hepatic TC also led us to examine other proteins involved in cholesterol uptake and transport in the liver. Hepatic protein levels of ABCG1 were decreased as well as that of the scavenger receptor SR-BI, which is involved in HDL uptake (Fig. S3A and S3B). Interestingly, both ACAT1 and ACAT2 which are enzymes involved in cholesterol esterification were elevated in mice treated with B[*α*]P (Fig. S3C and S3D).

**Figure 6:**
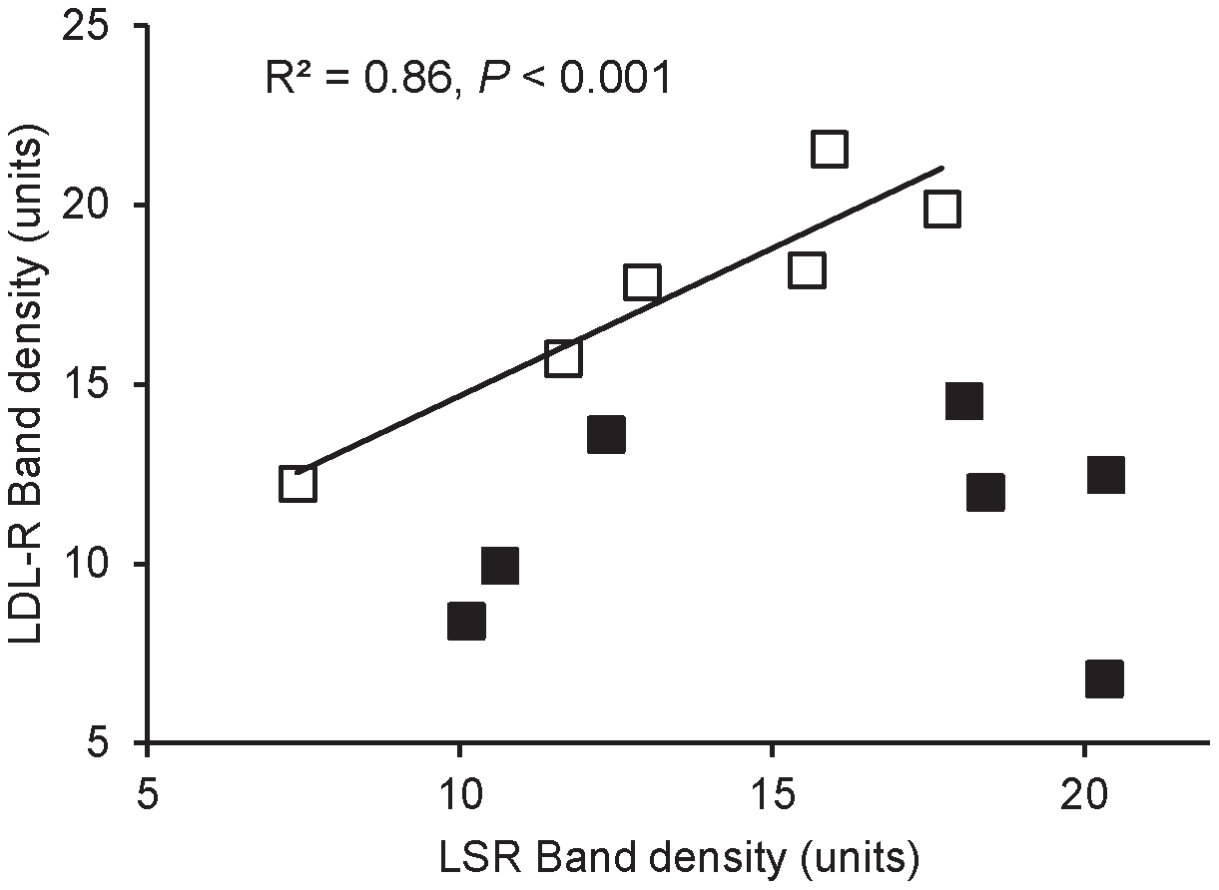
Scatter plot between hepatic LSR and LDL-R in mice. Distribution of individual values for liver membrane LSR and LDL-R protein levels are shown for mice from control (□) and B[*α*]P-treated (▪) groups.

LSR protein levels did not appear significantly different (Fig. 5A, left panel), while LSR mRNA was slightly but significantly higher in B[*α*]P-treated mice (Fig. 5B, left panel) As we had previously suggested a potential cooperativity between both receptors in the removal of TG-rich lipoproteins [8], and in view of the inter-individual variation of LSR protein observed in B[*α*]P-treated mice (Fig. 5A, left panel), protein levels for both LSR and LDL-R in each animal were compared on a scatter plot (Fig. 6). Interestingly, a significant positive correlation between hepatic LDL-R and LSR protein levels in control animals was observed. However, this correlation was no longer present in animals exposed to B[*α*]P, which led us to question if other potential correlations could be detected between plasma and tissue lipid parameters. Spearman rank correlation analysis was thus performed, and confirmed the positive correlation (Fig. 6) between LSR and LDL-R in control, but not B[*α*]P-treated animals (Table 2). Also, in B[*α*]P-treated mice, but not control animals, a positive correlation was detected between LDL-R and plasma TC, and negative correlations were observed between LDL-R and liver TC/PL ratio, as well as between LSR and liver TG/PL. ABCA1 protein levels were significantly correlated positively with plasma TG and negatively with liver TG/PL and TC/PL ratios, but again, only in B[*α*]P-exposed animals, and not in controls (Table 2). B[*α*]P exposure therefore appears to significantly modify relationships between LSR, LDL-R, or ABCA1 and various plasma and liver lipid levels, which would support the notion that exposure to B[*α*]P leads to dysfunction of the regulation of lipid homeostasis in these mice.

**Table 2:** Correlational analysis of lipid parameters measured in control and B[*α*]P-treated mice.

	LSR		LDL-R		ABCA1	
Parameter	Control^a^	B[*α*]P^a^	Control^a^	B[*α*]P^a^	Control^a^	B[*α*]P^a^
	Spearman correlation (ρ)					
**Plasma TC**	−0.42	−0.39	−0.31	0.82*	0.25	0.42
**Plasma TG**	0.10	0.54	0.60	0.71	0.00	0.80*
**Liver TC/PL**	−0.32	0.14	−0.50	−0.93**	0.64	−0.79*
**Liver TG/PL**	0.04	−0.75*	0.32	−0.61	−0.43	−0.82*
**LSR**	—	—	0.94**	0.10	0.21	0.57
**LDL-R**	—	—	—	—	−0.21	0.67
**ABCA1**	—	—	—	—	—	—

a n = 7 for each group.

* *P*<0.05, ** *P*<0.01, compared to control group.

### Effect of B[*α*]P on lipoprotein binding to receptors

**Figure 7:**
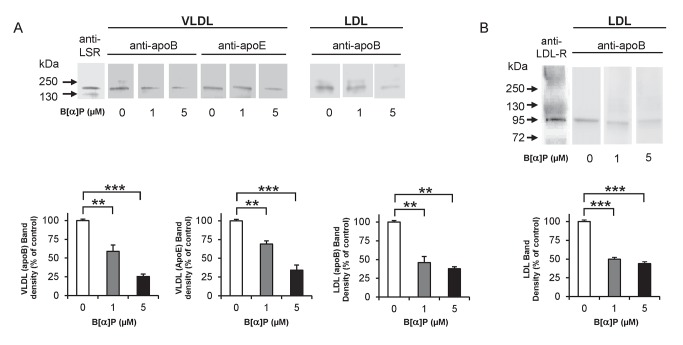
Effect of B[*α*]P on lipoprotein binding to (A) LSR and (B) LDL-R. Solubilized proteins from rat liver plasma membranes were separated on SDS-PAGE under non-reducing conditions and immobilized onto nitrocellulose membrane. For LSR, nitrocellulose membranes were pre-incubated with 0.8 mM oleate in order to activate the LSR complex. Strips were incubated at 37°C for 1 h with VLDL or LDL pre-incubated with 0, 1, or 5 µM B[*α*]P Strips were then washed and immunoblots were performed using anti-apoB or anti-apoE antibodies to identify LDL or VLDL, as indicated. Immunoblots using anti-LSR or anti-LDL-R antibodies were performed to verify the presence of the LSR complex or LDL-R (left strip for A and B, respectively). Densitometric analysis and representative blots are shown here of ligand blots performed on two different preparations of lipoproteins.

Since studies have shown that B[*α*]P in the circulation is found associated with lipoproteins [27], [28], the question arose as to whether B[*α*]P could directly affect the ability of lipoproteins to bind to LSR and LDL-R. To address this, we chose to use *in vitro* ligand blots, where a constant amount of receptor protein could be maintained, while exposing lipoproteins to different concentrations of the pollutant. The Ca^2+^-dependent (LDL-R) and -independent (LSR) binding properties, as well as apparent molecular weight of these two receptors allowed us to clearly distinguish the two receptors. Furthermore, LSR binds apoB- and apoE-containing lipoproteins (VLDL and LDL) only in the presence of oleate [7], [41], while this free fatty acid inhibits binding of LDL to its receptor [42]. Ligand blots were conducted using VLDL and LDL that had been pre-incubated 30 min at room temperature with 0, 1, and 5 µM B[*α*]P. LDL bound to the LDL-R or LSR was then detected by anti-apoB antibodies, while VLDL bound to LSR was detected using either anti-apoB or anti-apoE antibodies. The densities of bands revealing apoB or apoE bound to LSR (Fig. 7A) or LDL-R (Fig. 7B) became less pronounced with increasing concentrations of B[*α*]P, indicating a decrease in binding between the lipoprotein and LSR or LDL-R. Therefore, these results demonstrate that B[*α*]P can also directly interfere with the binding of the lipoprotein ligand to its receptor. The addition of 1 µM B[*α*]P on LDL and VLDL did not have any effect on their hydrodynamic radius (Table S1), nor on protein content, suggesting that this PAH did not change the structure of these lipoproteins. Similarly, incubation with 1 µM of B[*α*]P did not significantly change their approximate Zeta potential value (Table S1).

## Discussion

The objective of this study was to determine the effect of the common PAH pollutant B[*α*]P on lipid homeostasis in the liver. *In vitro* cell and ligand blot studies showed that B[*α*]P induced proteasome-mediated degradation of LSR, LDL-R and ABCA1, and also inhibited lipoprotein ligand binding to LSR and LDL-R, all of which could contribute to the changes in lipid status observed in mice exposed to this pollutant. Indeed, repeated treatment of B[*α*]P *in vivo* resulted in dyslipidemia in the form of increased plasma TC, TG, and TGRL, as well as increased liver TC content, and adipose tissue TG levels, the latter being compatible with the previously reported higher weight gain and fat mass.

Our results show that brief exposure of Hepa1-6 cells to <1 µM concentrations of B[*α*]P significantly diminished protein levels of LSR, LDL-R, as well as ABCA1. This effect was not related to a general cell loss since no significant changes were observed in the β-tubulin loading control, nor was cell death observed with the concentrations of the pollutant used (Supplementary Figure S1). Other PAH's including pyrene and phenanthrene did not display any effect on protein levels of LSR, LDL-R or ABCA1 when added to Hepa1-6 cells at similar concentrations, despite similar lipophilicities based on their octanol/water partition coefficients (log Kow for pyrene, 4.50-5.52 and for phenanthrene, 4.28-4.67) to that of B[*α*]P (log Kow 5.85-6.78) (H. Layeghkhavidaki,*et al*, unpublished data). This would therefore suggest that the B[*α*]P-induced decrease of these 3 proteins is specific to this pollutant. B[*α*]P has been shown to induce DNA adduct formation, however, only after prolonged incubations of at least 24 h [43], [44], while here, we used incubation times for ≤2 h, and with concentrations as low as 0.1 µM, 20-fold lower than those used for studies related to its carcinogenic properties [43], [44]. Furthermore, in the *in vivo* study, mice were exposed to concentrations of the pollutant that were 100-fold lower than those used to induce a tumorigenic response [45]. It is difficult to know precisely the levels of B[*α*]P to which individuals are exposed. This is a common pollutant formed by incomplete combustion of organic material, and can be inhaled, absorbed through the skin, and ingested. In the general population, different PAH levels range between 0.001 to 10 ng/mL [46]. In this study, the concentrations of B[*α*]P measured in plasma at the time of tissue sampling was measured at 0.69 ng/mL; this value was in the similar range as those found previously in plasma (0.04-1.62 ng/mL) from individuals in which this pollutant was found to be correlated to body mass index [19].

With regard to B[*α*]P metabolites, their toxico-kinetics in animal system are nowadays well established [47]. For instance, relative short half-lives (7.6 h to 9.2 h) have been described for 3-OH-B[*α*]P (which is often used as biomarker for assessing B[*α*]P exposure) following a single intravenous administration of B[*α*]P at 0.01 mg/kg and 0.05 mg/kg in rats [48]. The low level of rat exposure (0.5 mg/kg body weight of B[*α*]P every 48 h for 15 days) associated with the relative short half-lives of its metabolites may therefore explain why the concentration levels of all the metabolites analyzed 24 h after the last B[*α*]P administration were below the limit of quantification of the method used. Further studies would be needed to determine if metabolites of B[*α*]P and other PAH could exert similar effects.

Cell studies performed here showed that the proteasome was involved in the catabolism of LSR, LDL-R and ABCA1 in Hepa1-6 cells. This is actually the first evidence for proteasome degradation of LSR; proteasome involvement in the LDL-R and ABCA1 removal is consistent with previous work showing that the proteasome pathway can participate in part to the removal LDL-R and ABCA1 in HepG2 and macrophages, respectively [49], [50]. The more recently characterized PCSK9-mediated removal of LDL-R has also been shown to rely on the proteasome, but this may be cell-type dependent [51], [52]. Indeed, the lactacystin-induced increase of LDL-R appeared somewhat less pronounced as compared to that of LSR and ABCA1. B[*α*]P-induced reduction of the levels of these three proteins was practically abolished in the presence of the proteasome inhibitor, lactacystin, but not in the presence of the lysosomal inhibitor chloroquine, pointing towards proteasome-mediated removal as the mechanism underlying the effect of B[*α*]P. A previous observation showing that PAHs including B[*α*]P increase ubiquitylation of p21 protein in the A549 lung cancer cell line [53] lead us to suggest ubiquitylation as a potential mechanism by which this pollutant increased proteasomal degradation of the three proteins studied here. Additional investigation is required to determine if this represents increased degradation of newly-synthesized LSR or LDL-R, or vesicular LSR or LDL-R as a result of endocytosis from the plasma membrane.

Ligand blots using constant amounts of lipoprotein receptor revealed that the ability of lipoproteins exposed to B[*α*]P to bind LSR or LDL-R was significantly diminished. In a study in which B[*α*]P was added to plasma, apoB was found to be the major carrier of this pollutant [54]. LDL contains one apoB per particle, which is the apolipoprotein that is recognized by LDL-R or LSR. The presence of B[*α*]P on this apoB-containing lipoprotein could therefore interfere with the ability of LDL-R or LSR to recognize the binding epitope(s) of apoB, which may explain the observed decrease in binding. A similar effect could also explain the lower binding of VLDL to LSR. Binding of the apoE component of VLDL to LSR was also affected by B[*α*]P treatment, which may be due to direct interference in the binding of apoE to LSR, or simply to the indirect effect of reduced apoB-mediated binding of VLDL to LSR, since VLDL particles are large TGRL that contain both apoB and apoE. However, incubation of VLDL or LDL with 1 µM B[*α*]P did not lead to significant changes of the hydrodynamic radius and the approximate Zeta potential values obtained by dynamic light scattering and electrophoretic mobility measurements (Table S1), suggesting that B[*α*]P was adsorbed by hydrophobic interaction with the lipid component of the lipoproteins. Further biochemical studies are needed using purified receptor to determine the mechanisms by which this pollutant interferes with binding of the lipoproteins to these receptors.

We are unable to explain the differences in results for mRNA levels in the cell culture and animal studies. Despite this, the results based on cell culture and ligand blot studies obtained *in vitro* could provide a possible explanation for the changes in hepatic lipid metabolism reported in the *in vivo* experiments. In the liver of B[*α*]P-treated animals, LDL-R protein levels were significantly decreased, which may be due in part to a direct effect of the pollutant based on the cell culture studies, and/or as a result of the increase, albeit modest, in hepatic cholesterol content. Indeed, cholesterol acts as a sensor in the liver through the sterol regulatory element-binding protein pathway, which regulates hepatic needs for exogenous cholesterol by regulating LDL-R expression [55]. Since the absence of LDL-R expression is associated with increased plasma cholesterol and LDL [56], [57], the changes in LDL-R protein levels would be consistent with the increased plasma cholesterol measured. Direct interference of B[*α*]P with LDL binding to the LDL-R, as demonstrated in the ligand blots, also would contribute to increased plasma cholesterol. However, an increase in the plasma LDL fraction was not detectable in lipoprotein profiles obtained after separation by gel filtration, which may be due to the fact that LDL-R protein levels were reduced, and not completely absent.

ABCA1 was significantly diminished in the presence of B[*α*]P, in both the cell and animal studies. Indeed, this protein belongs to the family of multi-resistance drug transporters, which have been shown to be modulated through xenobiotic effects of B[*α*]P [32]. A previous study reported that B[*α*]P induced expression of ABCG1 after a 3-day exposure of Caco2 cells [58], however they used 50-fold higher concentrations than those used in this study with Hepa1-6 cells. Since ABCA1 plays a role in cholesterol efflux by providing cholesterol to lipid-poor apoA-I for secretion in the form of HDL, its reduced levels in B[*α*]P-treated mice may contribute to the observed increase in hepatic TC content. Indeed, significant negative correlations were observed between liver TC/PL ratio and ABCA1, but only in animals treated with B[*α*]P, and not in controls. A similar negative correlation was also observed between liver TG/PL ratio and ABCA1. A previous study showing that low ABCA1 levels are associated with non-alcoholic steatohepatitis and that siRNA-mediated knock-down of hepatic ABCA1 can lead to TG accumulation in the liver [59] would therefore be consistent with the negative correlations observed between liver lipid levels and ABCA1 in the B[*α*]P-treated mice.

The *in vitro* study suggests that B[*α*]P increased proteasome-mediated degradation of LSR. However, no significant changes were observed in hepatic LSR protein levels of B[*α*]P-treated mice. The fact that decreases in LDL-R and ABCA1 were nevertheless observed in B[*α*]P-treated mice suggests that LSR turnover rates may be different as compared to these other proteins; however, this remains to be verified. Ligand blots did demonstrate that B[*α*]P interferes with binding of VLDL and LDL to LSR. Since hepatic LSR is a receptor that plays a significant role in the removal of TGRL during the postprandial phase [6], [8], and taking into account that mice were injected with B[*α*]P during the postprandial period, this could explain the observed increase in plasma TG and TGRL. Indeed, radioactively-labelled B[*α*]P associated with chylomicrons is transported directly to the liver to be removed from the circulation [28]. We would therefore postulate that B[*α*]P-mediated inhibition of binding of TGRL to LSR at the site of the liver could contribute to the observed increase of TGRL in the circulation of B[*α*]P-treated mice.

Correlational studies revealed a strong positive correlation between LSR and the LDL-R, but only in the control group, which is consistent with a potential cooperativity between LSR and LDL-R regarding the removal of TGRL [8]. Interestingly, this correlation was no longer evident in B[*α*]P-treated mice. Furthermore, rather strong negative correlations were revealed between LSR and liver TG/PL, and between LDL-R and liver TC/PL, but only in the B[*α*]P group of mice. While this type of analysis cannot reveal causal relationships, it nevertheless supports the notion that B[*α*]P exposure can disrupt normal lipid homeostasis.

This study represents the first report on the direct effect of B[*α*]P on the hepatic lipoprotein receptors LDL-R and LSR. Increased plasma lipids have been shown in LSR^+/-^ mice [8] as well as in siRNA-mediated liver-specific inactivation of LSR mRNA [6], and in LDL-R^-/-^ mice [56], [57]. Interestingly, LSR^+/-^ exhibited higher weight gain as compared to controls with age, or if placed on a high-fat diet [10]. In addition, LDL-R^-/-^ mice placed on a high-fat and high-carbohydrate diet gained more weight as compared to controls [9]. This leads us to suggest that in a similar manner, B[*α*]P exposure *in vivo* leads to reduced functional LSR and LDL-R, which contribute to the changes in lipid status, as well as to increased susceptibility towards increased weight gain. Interestingly, we observed that levels of other hepatic proteins involved in cholesterol transport, SR-BI and ABCG1 were decreased in B[*α*]P –treated mice. On the contrary, protein levels of hepatic ACAT1 and ACAT2 involved in the synthesis of cholesteryl esters were actually increased, which may have contributed to the increased hepatic TC content observed in the mice exposed to this pollutant. These results would support the idea that besides LSR, LDL-R and ABCA1, other proteins involved in hepatic lipid and lipoprotein metabolism may also be affected by B[*α*]P and other pollutants. Additional studies are needed to explore the underlying molecular mechanisms. We have previously observed that B[*α*]P directly affects the ability of the adipocyte to release fatty acids after stimulation by adrenaline [20]. We have now shown in this study that this pollutant also contributes towards disrupting lipid homeostasis in the liver leading to dyslipidemia, thus revealing potential mechanisms that could explain part of the contribution of pollutants such as B[*α*]P to the etiology of dyslipidemia-linked obesity.

## Supporting Information

Figure S1 **Effect of B[** ***a*** **]P on cell viability.** Hepa1-6 cells were incubated at 37°C for 1 h with the indicated concentrations of B[*α*]P. Cell viability and metabolic activity tests were performed using (A) MTT, (B) calcein, and (C) trypan blue exclusion assays, as described in Materials and Methods. Results are shown as mean ± SEM of triplicate determinations.
(TIF)

Figure S2 **Time course effect of lactacystin on LSR, LDL-R and ABCA1 protein levels in Hepa1-6 cells.** Hepa1-6 cells were incubated at 37°C with 10 µM lactacystin for the indicated times. Immunoblots and densitometric analyses of the signal as a ratio to that of β-tubulin are shown for LSR, LDL-R and ABCA1 (*P<0.03, **P<0.01, compared to time 0, n = 3 per treatment).
(TIF)

Figure S3 **Effect of B[** ***a*** **]P treatment on hepatic protein levels of ABCG1, SR-BI, ACAT1 and ACAT2.** Immunoblots were performed to detect A) ABCG1, B) SR-BI, C) ACAT1 and D) ACAT2 protein levels in membrane (SR-BI) or cytosolic (ABCG1, ACAT1, ACAT2) fractions prepared from liver homogenates from mice treated with or without B[*α*]P. Densitometric analyses were performed, and are shown here as means ± SEM of n = 7 per group. Student's t-test was used to determine statistical difference (* *P*<0.05, ** *P*<0.01) as compared to control values.
(TIF)

Table S1 **Effect of B[** ***a*** **]P on the hydrodynamic radius and on Zeta potential of LDL and VLDL.**
(DOCX)
