# Supplementary figures and images for: Inhibitory Action of Benzo[α]pyrene on Hepatic Lipoprotein Receptors In Vitro and on Liver Lipid Homeostasis in Mice
Source: PLoS One. 2014 Jul 23;9(7):e102991. doi: 10.1371/journal.pone.0102991 (PMC4108373; doi:10.1371/journal.pone.0102991)

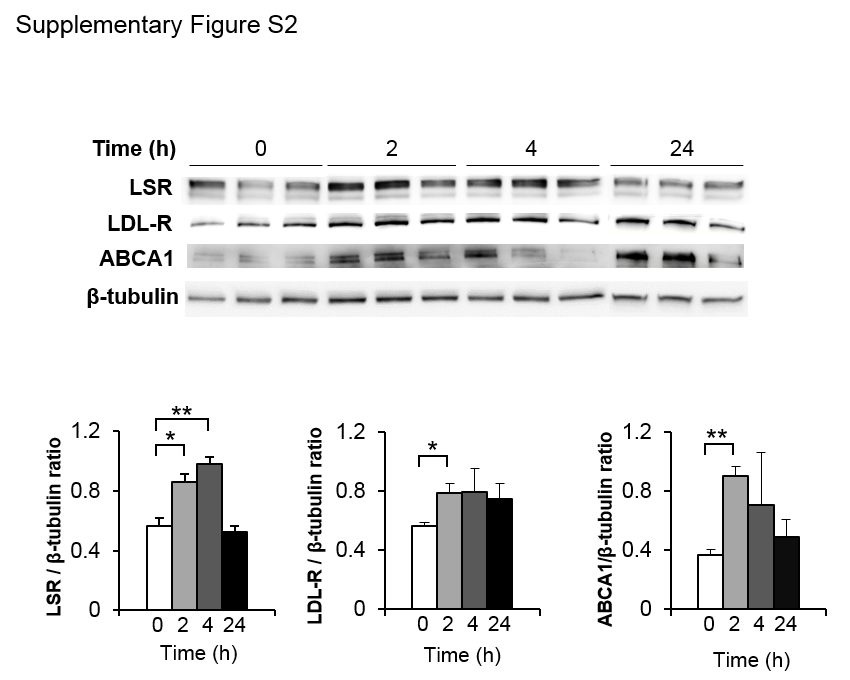

Supplement: Figure S2 — Time course effect of lactacystin on LSR, LDL-R and ABCA1 protein levels in Hepa1-6 cells. Hepa1-6 cells were incubated at 37°C with 10 µM lactacystin for the indicated times. Immunoblots and densitometric analyses of the signal as a ratio to that of β-tubulin are shown for LSR, LDL-R and ABCA1 (*P<0.03, **P<0.01, compared to time 0, n = 3 per treatment). (TIF) [file pone.0102991.s002.tif]

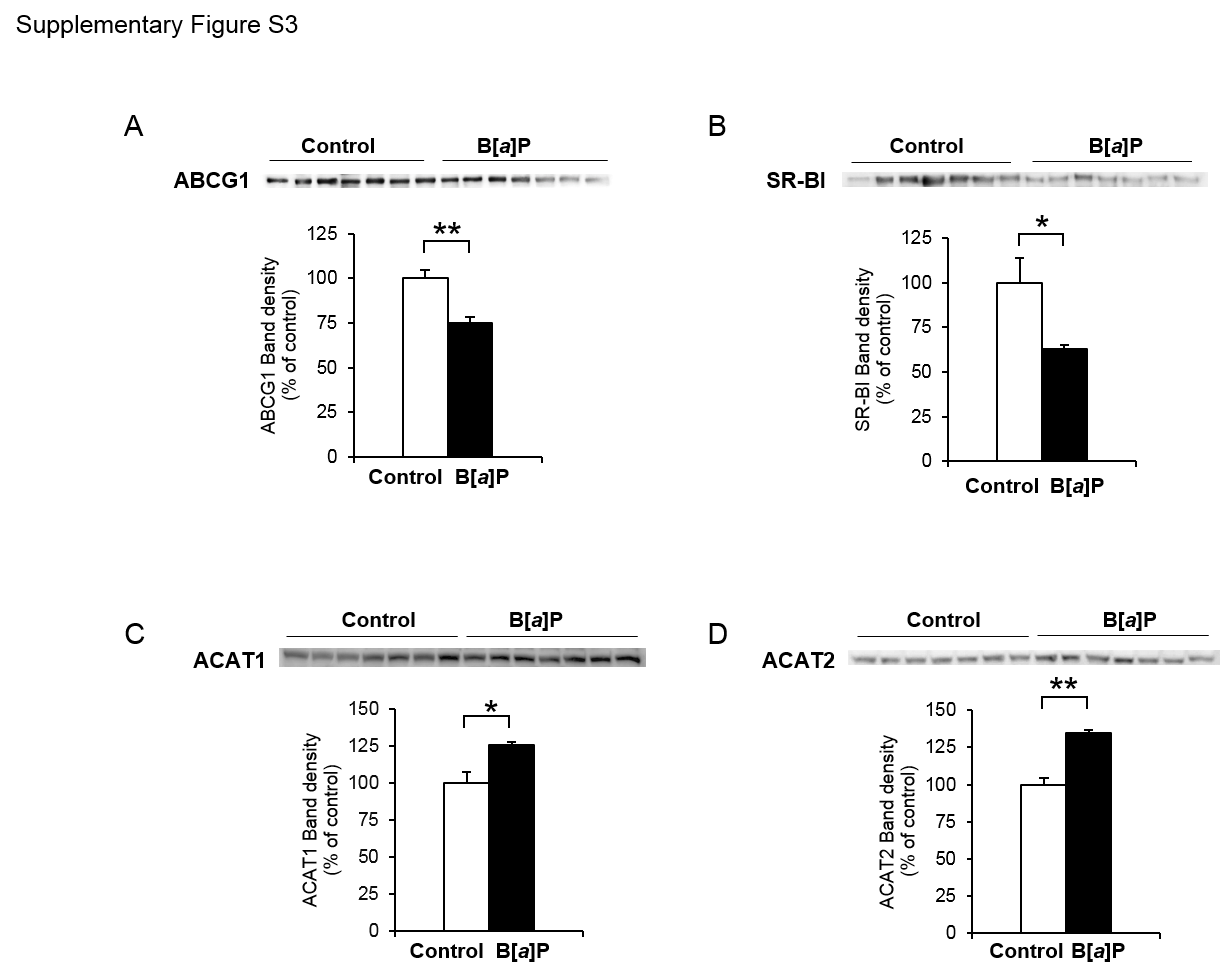

Supplement: Figure S3 — Effect of B[ a ]P treatment on hepatic protein levels of ABCG1, SR-BI, ACAT1 and ACAT2. Immunoblots were performed to detect A) ABCG1, B) SR-BI, C) ACAT1 and D) ACAT2 protein levels in membrane (SR-BI) or cytosolic (ABCG1, ACAT1, ACAT2) fractions prepared from liver homogenates from mice treated with or without B[α]P. Densitometric analyses were performed, and are shown here as means ± SEM of n = 7 per group. Student's t-test was used to determine statistical difference (* P<0.05, ** P<0.01) as compared to control values. (TIF) [file pone.0102991.s003.tif]
