# Supplementary material for: Inhibitory Action of Benzo[α]pyrene on Hepatic Lipoprotein Receptors In Vitro and on Liver Lipid Homeostasis in Mice
Source: PLoS One. 2014 Jul 23;9(7):e102991. doi: 10.1371/journal.pone.0102991 (PMC4108373; doi:10.1371/journal.pone.0102991)
Supplement: Table S1 — Effect of B[ a ]P on the hydrodynamic radius and on Zeta potential of LDL and VLDL. (DOCX) [file pone.0102991.s004.docx]

**Supplementary Table 1**. Effect of B[*a*]P on the hydrodynamic radius and on Zeta potential of LDL and VLDL.

|  | LDL | | | VLDL | | |
| --- | --- | --- | --- | --- | --- | --- |
| Benzopyrene (µM) | Z-average  R_h_ (nm) | PdI width (nm) | Approx. ζ (mV) | Z-average  R_h_ (nm) | PdI width (nm) | Approx. ζ (mV) |
| 0 | 12.3 | 5.3 | -28.6 ± 6.5 | 32.8 | 15.9 | -28.0 ± 13.0 |
| 1 | 12.4 | 5.3 | -28.0 ± 5.5 | 32.9 | 16.3 | -28.5 ± 10.2 |

VLDL and LDL were incubated with B[*a*]P as described for Figure 7 and measurements were made as described in Materials and Methods. Protein concentrations of VLDL and LDL before and after incubation with B[*a*]P were not significantly different. (R_h_: hydrodynamic radius; PdI: Polydispersity Index; Approx. ζ : Approximate Zeta potential).
